# Cecal Volvulus following a Right Nephrectomy for Wilms' Tumor: Should We Need to Close the Lateral Peritoneum?

**DOI:** 10.1055/s-0037-1612634

**Published:** 2018-01-08

**Authors:** Mauricio Gonzalez-Urquijo, Christian Ovalle-Chao, Eduardo Flores-Villalba, Ulises de Jesus Garza-Luna, Jose Humberto Velazco-De La Garza, Ulises Garza-Serna

**Affiliations:** 1 Department of Surgery, Escuela de Medicina, Tecnologico de Monterrey, Monterrey, Nuevo Leon, Mexico; 2 Department of Paediatric Surgery, Universidad Autonoma de Nuevo Leon, San Nicolas de los Garza, Nuevo Leon, Mexico

**Keywords:** cecal volvulus, lateral peritoneum, nephrectomy, Wilms' tumor

## Abstract

Wilms' tumor (WT) accounts for 90% of all pediatric renal malignant tumors. The most common postoperative complication based on the National Wilms' Tumor Study is small bowel obstruction. We report on a 2-year-old girl with postoperative bowel obstruction following a right nephrectomy for WT. The patient was reintervened 48 hours after surgery and a cecal volvulus was found. Here, we will describe possible causes of this postoperative complication and discuss management.

## Introduction

Wilms' tumor (WT) is the most common childhood malignant renal tumor, it represents almost 90% of pediatric renal tumors and 7% of all childhood malignancies, occurring mainly in patients younger than 5 years of age and it has a 5-year survival rate of 85%. 1 2

Surgical complications based on the National Wilms' Tumor Study (NWTS) protocol with an upfront primary nephrectomy has significantly decreased over the past decade with postoperative bowel obstruction of the small intestine being the most common. 3

Large bowel volvulus is a rare cause of bowel obstruction occurring mainly in pediatric patients, with the cecum being the most common location in some series and it represents less than 1% of all intestinal obstructions in children. 4 5 6 7 Although postoperative cecal volvulus following a nephrectomy in adult patients 5 8 9 10 have been described previously, to our knowledge, there are no reported cases of a cecal volvulus following a nephrectomy in children and more specifically after a WT resection.

We present the case of a 15-month-old female patient with a cecal volvulus following a right nephrectomy of a WT.

## Case Report

**Fig. 1:**
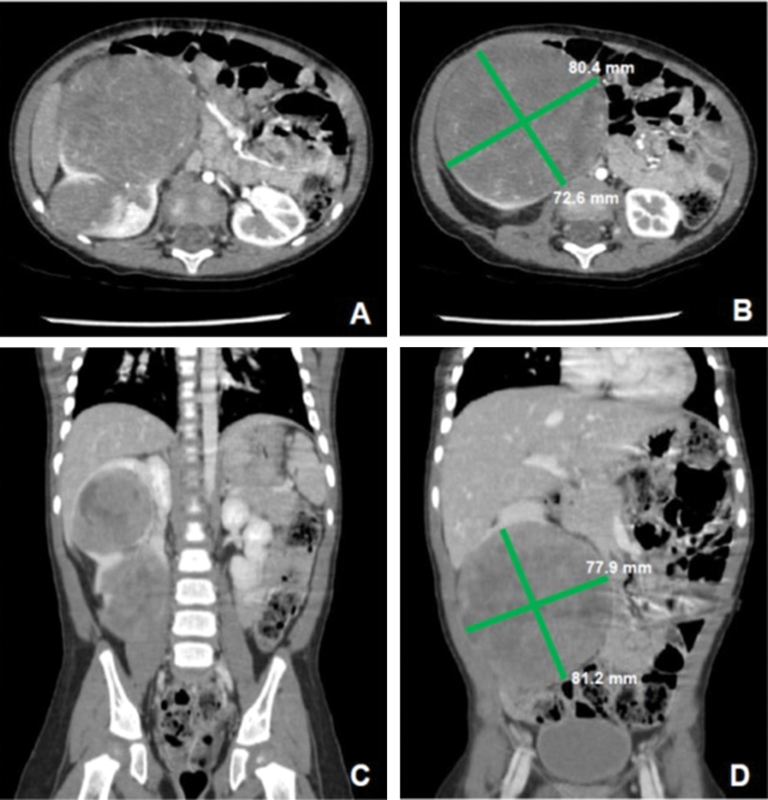
Abdominal computed tomography scan with intravenous contrast. (A, B) (Superior to inferior cuts) axial view; (C, D) (posterior to anterior cuts) coronal view; 8 × 7 cm right kidney tumor.

A 15-month-old female patient was brought to our teaching hospital and was admitted through the emergency room following a nonpainful abdominal distention and a right abdominal mass. An ultrasound confirmed the presence of a right renal mass, and a computed tomography (CT) scan showed an 8 cm × 7 cm right renal tumor; no thrombosis of the renal or inferior vena cava was observed ( Fig. 1 ). A chest CT scan showed no evidence of pulmonary metastasis. The patient was then scheduled for a right nephrectomy.

**Fig. 2:**
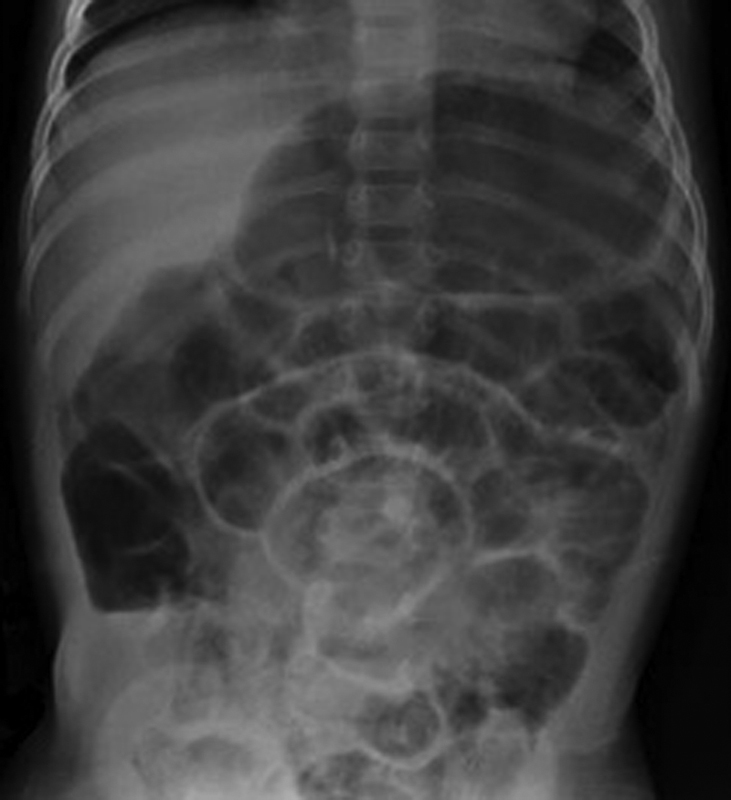
Abdominal X-ray. Right lower quadrant fixed air fluid level and small bowel dilatation.

**Fig. 3:**
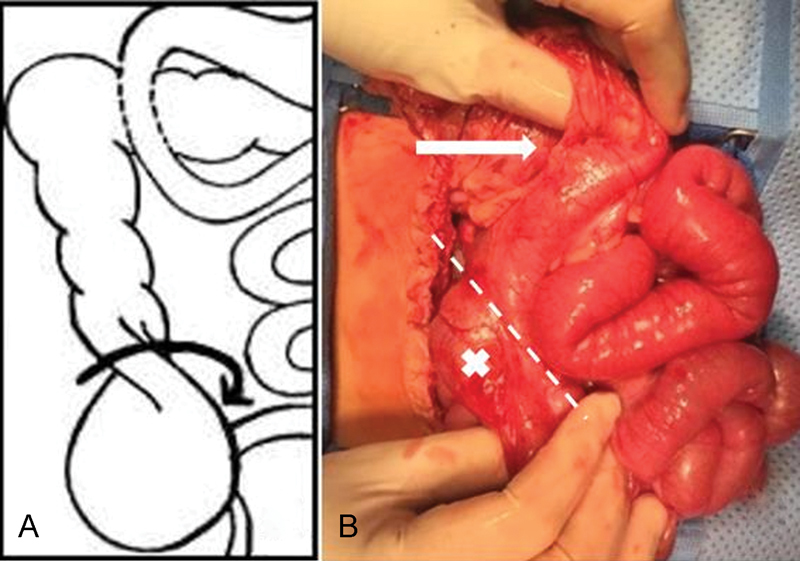
(A) Scheme demonstrating the cecal volvulus found during reintervention. (B) Solid arrow showing medial mobilization of right colon. Dashed lined showing the transition point of obstruction after detorsion of the cecum (“x” mark).

For a better longitudinal exposure of the vena cava, we entered through an incision in the midline, immediately displacing the cecum and the right colon medially. The right lateral peritoneum was divided to allow access to the renal tumor and finally the right kidney was removed along with a para-aortic lymphadenectomy. The right colon and the small bowel were left in place inside the abdominal cavity during the entire procedure, and we did not close the lateral peritoneum before closing the abdominal wall. Forty-eight hours after surgery, the patient showed abdominal distention and one episode of bilious vomiting. An abdominal X-ray showed the presence of fixed right lower abdominal air fluid levels and showed small bowel dilatation ( Fig. 2 ). The patient was taken to the operating room for an exploratory laparotomy performed through the previous incision. The small bowel was intact, and a cecal volvulus was found ( Fig. 3 ). A detorsion was performed showing a viable bowel and the lateral peritoneum was closed. The patient had an uneventful recovery and was discharged on postoperative day 5. Pathology confirmed a multifocal right renal stage I WT.

## Discussion

Based on the NWTS protocol, nephrectomy is the primary upfront treatment for WT. Preoperative chemotherapy is reserved for patients who have a solitary kidney, a horseshoe kidney, thrombosis of the inferior vena cava above the level of the hepatic veins, or if the child presents with respiratory distress due to extensive pulmonary metastases. 11 12 13 Based on the CT scan, our patient did not meet the criteria for preoperative chemotherapy according to the NWTS protocols; therefore, we decided to perform a nephrectomy.

Several postoperative complications of primary nephrectomy following the NWTS protocol approach have been described, with bowel obstruction being the most common appearing in 5.1 to 6.9% of the patients, compared with 2.5% of the patients following the Societe Internationale D'oncologie Pediatrique protocol; postoperative adhesions followed by intussusceptions and internal hernias have all been described. 3 14

Postoperative intussusception usually occurs within the first postoperative week with a mean of 5 days; 15 16 bowel adhesions in infants and children causing bowel obstruction usually have a late presentation with 66% of them presenting 1 year after the initial procedure in some series, and only 6.8% of them being secondary to the resection of WT. 17 Internal hernias following a nephrectomy with small bowel obstruction usually have an acute presentation and can be either transmesenteric or in the retroperitoneal fossa. 14 18 19 20

An important differential diagnosis of patients presenting with volvulus is malrotation. This entity should always be kept in mind when assessing any infant or child with symptoms of vomiting and pain, particularly when the vomiting is bile stained. Ultrasound examination may be helpful but is not secure enough to exclude the diagnosis. Laparotomy or laparoscopy is the only way to be sure. 21

From the several risk factors identified that can increase the rate of postoperative surgical complications following the resection of a WT (including higher local tumor stage, intravascular extension, and en bloc resection of other visceral organs 3 ), none of them has been reported as a specific risk factor for postoperative bowel obstruction. 22

A cecal volvulus following a right nephrectomy in adults has been described previously after releasing the cecum from the peritoneum and positioning it medially, 5 9 10 although it has also been described following a left-sided nephrectomy. 8

The following have been described as options for cecal volvulus management: cecopexy, simple detorsion without fixation, detorsion with cecopexy, tube cecostomy, and ileocolectomy. 23 There is no consensus on which is the best option for the management of the cecal volvulus, but there is an agreement between series that the management should rely on the viability of the bowel. 24 25 26 Detorsion might be sufficient for patients with a viable bowel, without gangrene, and a cecostomy has had a higher rate of reported complications in almost half of the series, with recurrence in approximately 15% and mortality rates of 20%. 23

We decided to do a detorsion of the cecal volvulus because the bowel was viable; even though we do not usually close the lateral peritoneum following a nephrectomy for WT, we decided to close the lateral peritoneum on the second intervention as a safety measure to keep the colon in its place.

A volvulus of the cecum should be considered as a possible cause of intestinal obstruction in the postoperative period following a right nephrectomy for WT, with a surgical intervention being the treatment of choice. The management of a cecal volvulus should be decided based on the viability of the bowel. A simple detorsion might be sufficient for the successful management of the condition, but we believe that closing the lateral peritoneum helped minimize the movement of the right colon during our second intervention. Although based on the evidence we cannot conclude that closure of the lateral peritoneum is mandatory, we do believe that this might have played a huge role in the effectiveness of the procedure.

## New Insights and the Importance for the Pediatric Surgeon

When performing a nephrectomy it might be important to consider closing the lateral peritoneum as it might play a role in avoiding possible post-operative cecal volvulus.
